# Apathy, Cognitive Impairment, and Social Support Contribute to Participation in Cognitively Demanding Activities Poststroke

**DOI:** 10.1155/2021/8810632

**Published:** 2021-03-26

**Authors:** Amy Ho, Marjorie L. Nicholas, Chaitali Dagli, Lisa Tabor Connor

**Affiliations:** ^1^Department of Occupational Therapy, MGH Institute of Health Professions, Boston MA, USA; ^2^Department of Communication Sciences & Disorders, MGH Institute of Health Professions, Boston MA, USA; ^3^Program in Occupational Therapy, Washington University School of Medicine, St. Louis MO, USA; ^4^Department of Neurology, Washington University School of Medicine, St. Louis MO, USA

## Abstract

**Objective:**

To understand the extent to which apathy, cognition, and social support predict participation in activities with cognitive demands.

**Design:**

Prospective, quantitative correlational, cross-sectional study. *Setting*. Outpatient treatment centers and community stroke support groups located in St. Louis, MO, and Boston, MA. *Participants*. 81 community-dwelling individuals ≥ 6-month poststroke with and without aphasia. *Measures*. Participants completed the Activity Card Sort (ACS), Apathy Evaluation Scale (AES), Medical Outcomes Study Social Support Survey (MOS-SSS), and Delis-Kaplan Executive Function System (DKEFS) Design Fluency and Trail-Making subtests.

**Results:**

Cognitive deficits limit participation in activities with high cognitive demands. Apathy and positive social interaction influence participation, regardless of high or low cognitive demands. Poststroke aphasia did not impact return to participation in activities with high and low cognitive demands. *Conclusions and Relevance*. Cognitive deficits seen poststroke contribute to participation only for activities with high cognitive demands. Apathy has a significant and negative influence on participation overall. Social support is a modifiable contextual factor that can facilitate participation. Poststroke apathy can be detrimental to participation but is not well recognized. The availability of companionship from others to enjoy time with can facilitate participation.

## 1. Introduction

Stroke is the leading cause of complex disability in the United States with an additional 3.4 million US adults projected to have a stroke by 2030 [1]. Stroke leads to lasting impairments in physical, emotional, cognitive, and language domains [2–4] that can negatively impact long-term participation, resumption of meaningful occupations, and quality of life (e.g., [5, 6]). To individuals, stroke recovery is synonymous with resuming participation in meaningful prestroke activities and reintegrating into normal living [7]. Over 65% of stroke survivors, however, report participation restrictions in reintegrating into normal living six months into their recovery. Additionally, three-quarters of individuals poststroke do not occupy their day with any meaningful social, leisure, or occupational activities [8]. Moreover, participation limitations persist for years after stroke [9, 10].

Postacute stroke rehabilitation is aimed primarily at improving functional independence in self-care activities, placing a major focus on physical and functional recovery. However, the emphasis on physical sequela often overshadows other areas of deficit that have a negative impact on long-term participation outcomes such as cognitive and motivational consequences of stroke. For instance, over the course of three years, Kapoor et al. [11] followed individuals who had physically recovered poststroke and were independently performing their basic ADLs. Despite having physically recovered, one-third of their participants endorsed depressive symptoms and over half experienced cognitive impairments and participation restrictions. While physical function and self-care are important components of participation, addressing stroke recovery with the goal of enabling long-term participation outcomes is imperative and extends beyond functional independence for physical activities.

Although restoration of independent performance in ADLs may be realized, participation in instrumental activities of daily living (IADLs) and other complex recreational, occupational, and social activities often lags behind [8]. More specifically, stroke survivors report limitations with their ability to prepare meals, manage their finances, shop, drive, and fully participate in education, vocational activities, and indoor and outdoor leisure activities [8–10, 12]. This discrepancy between ADL and IADL performance poststroke may be explained by greater activity requirements of IADLs, specifically dependence on higher-order cognitive skills such as executive abilities.

Individuals poststroke report dissatisfaction in their participation with activities requiring cognition [12]. Rudimentary activities are primarily physically demanding, relying on habitual, motoric performance. Complex activities are much more cognitively demanding, requiring active problem-solving and organized planning [13, 14]. Complex activities are not limited to the home; they also include activities in the community. Community activities may require cognitive skills supporting awareness of the dynamic environmental context and adaptation to new situations. The ability to participate in complex activities is necessary for independent living in the home and community [9, 13], warranting a closer investigation into cognitive demands involved in complex tasks. Yet, there is a paucity of stroke studies that examine activity demands to understand the role they may play in restricting participation.

In addition to examining demands of activities themselves, it is important to consider the cognitive and motivational capabilities of the individual that are required to participate in complex activities. Understanding goal-directed behavior that precedes performing complex activities is necessary to understand how cognitive and motivational limitations may impact participation in activities with high cognitive demands. The ICF [15] identifies motivation and executive function as essential for goal-driven behavior. Higher-level cognitive functions such as executive abilities are thought to be foundational prerequisites to successfully complete daily complex tasks, vocational responsibilities, leisure activities, and social participation (e.g., [16]). Stroke survivors who experience executive function deficits are less likely to resume participation in complex IADLs, social roles, leisure activities [17, 18], and work [19].

Apathy is a neuropsychiatric consequence of a stroke presenting as decreased motivation, emotional detachment, and decreased engagement in previously preferred activities, thereby resulting in reduced goal-directed behavior [20, 21]. An estimated 39% of stroke survivors experience cognitive impairments [22] and 34% experience apathy [23]. However, a scarcity of stroke studies examines both higher-level cognitive abilities and apathy with respect to participation in complex tasks among stroke survivors.

The reduction of goal-oriented activity in people with apathy is thought to have affective, behavioral, and cognitive components [20, 24]. The motivational and emotional disturbances brought on by apathy may be amplified by deficits in cognitive performance [25]. Furthermore, apathy impedes rehabilitation participation [23], resulting in reduced engagement in intense early rehabilitation programs and consequently worse functional and cognitive performance outcomes [21, 24, 26]. These collective cognitive and motivational problems and the participation limitations in meaningful activities that result negatively impact stroke survivors' quality of life [6].

Roughly one-third of strokes result in aphasia, an impairment impacting language comprehension, expression, or both [27]. The prevalence of apathy is twice as high in stroke survivors with aphasia as those without [3]. Stroke survivors with aphasia are inconsistently reported to experience more severe cognitive deficits in executive function, memory, and attention, than those without aphasia [4, 28]. The uncertainty regarding concomitant cognitive deficits may be due to difficulty in disentangling the contributions of language deficits and nonlinguistic cognitive deficits [29]. This complexity, along with the difficulty of managing the communication impairment, often results in people with aphasia (PWA) being excluded from stroke studies (e.g., [30]). However, with the proper adaptations of assessment materials and communication accommodations, PWA can participate in stroke studies [31, 32]. Given these findings that individuals with aphasia may be disproportionately affected by apathy and inconsistently by cognitive deficits, further investigation regarding the retention of prestroke cognitively demanding activities is warranted.

Environmental contextual factors, such as availability of social support, can facilitate meaningful participation, despite deficits in cognitive, motivational, and communication skills. Social support is typically conceptualized by structural or functional social support [33]. Structural social support objectively measures the quantifiable breadth of existing social connections, whereas functional social support subjectively measures the robust interpersonal relationships that are readily available to meet one's needs and serve a supportive purpose. In mitigating stroke-related stressors, social support has been shown to positively predict social and community participation [34, 35]. Three recent systematic reviews examining the determinants of poststroke participation found that social support was a common contextual facilitator for participation [36–38]. Moreover, in population-level studies focusing on healthy aging adults, social support was found to help preserve cognitive functioning, such as executive functioning and memory [39–41]. Empirical evidence suggests social support is a modifiable contextual factor that may preserve cognitive functioning of healthy aging adults and serves as a facilitator for participation after a stroke event; however, limited studies have investigated the generalizability of these benefits to poststroke participation in activities with cognitive demands.

Studies support the unique participation restrictions that stroke survivors experience [2, 7, 42]. Gadidi et al. [10] found that functional ability predicts activity limitations, which in turn predict participation restrictions in chronic stroke survivors. To better understand participation restriction, we must critically assess the demands of the activities themselves and the cognitive, motivational, and language capabilities that the person with stroke has to determine whether participation can be explained by the match between person-specific factors and the demands of particular activities and whether social support can help to mitigate participation restrictions.

Evidence suggests poststroke cognitive dysfunction, apathy, and aphasia limit participation and that the cognitive task demands of complex activities can pose as barriers to participation. Furthermore, social support may facilitate participation despite these health consequences. The aim of this study is to understand the extent to which apathy, cognition, and social support facilitate or hinder participation in activities with high and low cognitive demands among community-dwelling stroke survivors with and without aphasia.

## 2. Method

This research was approved by the Partners Healthcare Institutional Review Board and the Washington University Human Research Protection Office with participants providing written informed consent. The human study processes and the consent procedures conformed to the Declaration of Helsinki, 1964.

### 2.1. Participants

Participants were recruited from the Cognitive Rehabilitation Research Group Stroke Registry at Washington University in St. Louis, the IMPACT Practice Center at MGH Institute of Health Professions in Boston, and local stroke support groups in the Greater Boston metropolitan area. The study recruited stroke survivors, with and without aphasia, who were living in the community and met the following inclusion criteria: at least 18 years old, first stroke, at least six-month poststroke, able to commute to the testing location, and able to withstand two testing sessions that lasted two to three hours each by self-report. Individuals with aphasia needed to have a diagnosis of aphasia and the ability to reliably answer aphasia-adapted questions requiring a yes or no response. Yes/no responses were assessed with questions regarding key elements of the consent form. Aphasia-adapted questions about key components of the study were presented in written form and were read to participants for a response. For example, “Are we doing this study to learn about a new drug?” We expected the participant to answer the 7 questions correctly after no more than one opportunity for explanation in order to be enrolled in the study. Exclusion criteria consisted of a history of additional strokes or a history of nonstroke-related physical, cognitive, neurological, or psychological disorders including an ongoing seizure disorder. Demographic information of the 81 enrolled participants is displayed in Table 1.

### 2.2. Data Collection and Outcome Measures

Measures were adapted to facilitate communication for all participants while preserving the psychometric integrity of various assessments [32]. Using a multimodal approach, the administration, presentation, and response format of the assessments were adapted to support reading and auditory comprehension, though the wording of the items was not altered. Applying the principle of supported communication ramps [31], a systematic hierarchy of examiner supports was utilized when indicated. Participants were administered a battery of assessments during two sessions requiring approximately 5 to 6 hours to complete. The assessments included in this investigation are a subset of the total assessment battery.

#### 2.2.1. Participation

The Activity Card Sort (ACS) measures participation retention among 89 activities, which comprise 20 instrumental, 35 social, 17 high physically demanding, and 17 low physically demanding leisure activities [43]. Each activity is represented on an individual card by a corresponding photograph and caption. Participants placed the individual cards into structured categories that represent previous and current participation. Scores were then calculated to indicate the percentage of prestroke activities retained. The ACS has been demonstrated to have high internal consistency, high test-retest reliability, and content, construct, and predictive validity (e.g., [44]).

To categorize ACS activities along a cognitive continuum, data from an unpublished normative study was used. Healthy adults (*N* = 43) rated the extent (none = 0, some = 1, fair = 2, a lot = 3) to which each activity required 9 different demands to participate, including cognitive demands, the dimension of interest to the current study. The calculated average ratings (little = 0-0.99, fair = 1.0-1.99, a lot = 2.0-3.0) for each activity were used to group them into the respective activity demand categories. For the specific dimension of activities requiring cognitive skills, 27 activities were identified as requiring a lot of cognitive skill (high CS), that is, activities with an average rating ≥ 2.0, and 14 activities were identified as requiring little to no cognitive skill (low CS), that is, activities with an average rating < 1. Notably, each of these subsets that differed in cognitive demand had about the same number of items from the low demand leisure and the high demand leisure categories from the ACS. The high CS subset did have more IADL items than the low CS subset (see Tables 2 and 3 for specific items), but percent retained was the outcome measure used, not number of items retained. There were 48 activities with middle-range cognitive scores not considered in this analysis.

#### 2.2.2. Apathy

The Apathy Evaluation Scale (AES) measures self-reported apathy symptoms within the last four weeks on a 4-point Likert scale [20]. Total summed scores range from 18 to 72 with higher scores indicating more apathy symptoms. For the post hoc analysis, an AES cutoff score of ≥37 was selected to indicate apathy [24, 45]. The psychometric properties of the AES indicate excellent internal consistency, high internal reliability, excellent interrater reliability, and a test-retest reliability of 0.76-0.94 [20].

#### 2.2.3. Cognition

The Delis-Kaplan Executive Function System (D-KEFS) is a neuropsychological assessment that measures executive function [46]. Two of the nine subtests were utilized, the Trail Making (TM) Test and Design Fluency (DF) Test. The composite D-KEFS score used in the analysis was the average of the scaled scores from the Trail Making subtest, condition 4—number-letter switching, and the Design Fluency subtest, condition 3—switching. The D-KEFS has high internal consistency and moderate test-retest reliability [46].

#### 2.2.4. Social Support

The Medical Outcomes Study Social Support Survey (MOS-SSS: [47]) is a self-reported measure of functional social support in four domains: tangible, affectionate, emotional-informational, and positive social interaction, using a 5-point Likert scale with higher scores indicating more social support. The MOS-SSS has high internal consistency and good validity and reliability [48]. Based on prior work from our laboratory, the MOS-SSS positive social interaction score was included as a potential predictor [35].

### 2.3. Data Analysis

Data analysis was conducted with SPSS Statistics 24.0 [49]. First, an independent samples *t*-test was utilized to identify whether there was a significant difference between persons with aphasia (PWA) and persons without aphasia (PWOA) on the primary outcome variables—the percent retained low-CS activities and percent retained high-CS activities as measured by the ACS. If significantly different, aphasia status would be included as a variable in the regression models. Second, bivariate Pearson correlations were calculated to inform which variables to include into the hierarchical regression analysis. Variables that significantly correlated (*p* < 0.05) with either of the two outcome measures were included in the regression model. Third, two hierarchical regression analyses were conducted to determine how much of the variance in percent retained low-CS and high-CS activity scores was accounted for by the predictor variables. Last, a post hoc analysis compared differences in participation scores and predictor variables in those with and without apathy.

## 3. Results

Of the 81 participants, 43 were PWA and 38 were PWOA. The average age of the entire sample was 60 years old with participants averaging 5-year post stroke with mild stroke severity scores at the chronic stage. The results of the independent samples *t-*test showed no significant differences between the two groups in retention percentages for low- (*t*(79) = −0.465, *p* = 0.644) and high-CS activities (*t*(79) = 0.923, *p* = 0.359), displayed in Table 4. Therefore, aphasia status was not retained as a variable in the regression analyses.

The potential predictor variables and their correlations with percent retained participation in activities with low and high cognitive demands are shown in Table 5. There was a positive correlation between cognition scores and percent retained low-CS activities and between MOS-SSS positive social interaction scores and percent retained low-CS activities, such that higher cognitive ability and greater social support were associated with higher percent retained low-CS activities. Similarly, there were significant positive correlations between cognitive skill and social support and percent retained for high-CS activities. Apathy scores were significantly and negatively correlated with both categories of CS activities, such that the greater the apathy score, the lower the percent retained for both high-CS and low-CS activities.

The top half of Table 6 represents the results of the multiple linear regression analysis examining predictors of retained participation in low-CS activities. A significant regression model was obtained (*F*(3, 77) = 12.9, *p* < 0.0001) with predictor variables accounting for 33.4 percent of the variance in low-CS activities. As expected, the cognition composite score was not a significant independent predictor of low-CS activity retention. Both MOS-SSS positive social interaction and AES were statistically significant independent predictors of percent retained low-CS activities.

The bottom half of Table 6 represents the results of the multiple linear regression analysis examining predictors of retained participation in high-CS activities. A significant regression model was obtained (*F*(3, 77) = 16.7, *p* < 0.0001) with predictor variables accounting for 39.4 percent of the variance in high-CS activities. Cognition, as predicted, was an independent predictor of high-CS activity retention, in contrast to the results for the low-CS activity retention model. Positive social interaction and apathy were also statistically significant predictors of percent retained high-CS activities.

Apathy was a significant predictor in both models for retained participation in activities with low-CS and high-CS activities. To investigate the extent to which individuals with and without clinically significant apathy differed in their cognitive, social support, and participation level, a post hoc analysis of apathy was implemented (see Table 7). Participants with AES scores < 37 or ≥37 were identified as nonapathetic and apathetic, respectively [24, 45]. The results of the independent samples *t-*tests showed there were significant differences between the groups in retained low-CS (*t*(79) = −3.68, *p* < 0.0001, *d* = −0.890) and high-CS activities (*t*(79) = 3.612, *p* = 0.001, *d* = 0.907) and overall perceived social support (*t*(79) = −4.037, *p* < 0.0001, *d* = −0.995) such that people who met the clinical cutoff for apathy had lower activity retention for high-CS and low-CS activities and fewer positive social interactions.

A post hoc analysis was conducted to determine if motor impairment, as measured by the NIHSS Total Motor Items, could account for differences in those with and without apathy. There was, however, no significant correlation of NIHSS Total Motor Items with the AES (*r*(71) = .107, p = 0.367), nor did those with significant apathy differ from those without significant apathy with regard to their NIHSS Total More Item score (*t*(71) = 0.885, *p* = 0.379, *d* = 0.236).

## 4. Discussion

Our study sought to understand the extent to which apathy, cognition, and social support predict participation in activities with high versus low cognitive demands as measured by the ACS. Nearly 40% of the variance in percent retained high-CS activities was explained by apathy, cognitive abilities, and positive social interaction. For percent retained low-CS activities, the same predictive variables explained 33% of the variance. Although there is a considerable proportion of variance that is unaccounted for in these activities, the findings point out that apathy, cognition, and social support all play a role in the retention of prestroke activities. These factors may provide avenues for intervention to increase participation via remediation, compensatory strategies, modifying the activities themselves, or modifying the environment in which the activities are done.

Notably, participants who were nearly 5-year postmild stroke living in the community gave up, on average, 33% of their high-CS prestroke activities and 20% of their low-CS prestroke activities. That translates to a loss of approximately 9 activities in the high-CS domain and approximately 3 activities in the low-CS domain. This degree of restriction appears remarkable in a group of people who experienced only a “mild” neurological event. This pattern of a greater number of activities given up in the high-CS domain provides beginning support for the hypothesis that individuals who experience stroke may have cognitive deficits that have an impact on participation in cognitively demanding activities. We did not, however, detect a difference in the percentage retained of high- and low-CS activities between PWA and PWOA. Thus, the presence of mild aphasia did not differentially impact retained participation in activities with low or high cognitive demands, suggesting that participation in activities with cognitive demands can be explained by factors other than language ability. It is possible that a sample with more severe aphasia might have shown different effects on retention of participation in cognitively demanding activities.

Perceived positive social interaction correlated with participation in low-CS and high-CS activities. Moreover, positive social interaction was a significant independent predictor of participation in both low CS and high CS. This suggests that having the companionship of a supportive individual to enjoy activities with facilitates participation in activities with or without cognitive demands. Perhaps, the perceived support of having someone to engage in activities with is cognitively stimulating [41] and encourages participation in a variety of activities, regardless of cognitive demands. The findings in this study are consistent with previous work from our lab [35] showing that positive social interaction is an independent predictor of social participation. Addressing modifiable contextual factors, such as social support, may be an effective way to augment participation outcomes.

Objectively derived estimates of cognitive skills have differential predictive power depending on the cognitive complexity of activities. This finding supports the assertion in the literature that executive functions and higher-order cognitive functions are necessary for participating in complex activities (e.g., [16]; WHO, 2011). Similarly, Reppermund et al. [14] found that among individuals with mild cognitive impairments, better cognitive ability outcomes related to more involvement in daily activities with high cognitive demands. Cognitive skills underlie a range of activities, including IADLs, social participation, and leisure activities (see Tables 2 and 3). Thus, by addressing participation in activities with high cognitive demands, the goal would be achieved to target participation in activities beyond self-care that are meaningful to clients.

Apathy was found to be both significantly correlated with and independently predictive of both low-CS and high-CS activity retention. Apathy is a mood and motivation disruption that inhibits goal-oriented behaviors, affecting the initiation and execution of once meaningful activities. The association of apathy with restriction in participation of activities in people with chronic stroke is similar to the finding of Babulal et al. [25], namely, that apathy was predictive of the need for increased cognitive support with initiation, organization, sequencing, safety and judgement, and ability to execute to completion complex daily tasks in individuals within the first 3 months poststroke.

In our post hoc analysis focused on clinically significant levels of apathy, apathy was a significant barrier for return to prestroke baseline. Individuals with apathy experienced decreased participation in activities, regardless of cognitive demands and felt less supported by their social network compared to individuals without apathy. Nearly one-third of our sample reported clinically significant apathy, which is in line with the estimated prevalence of 34% found in other stroke studies [23]. Yet, poststroke apathy remains underrecognized. The annual statistical update on stroke from the American Heart Association does not include apathy in their report [1], despite its ubiquity and disruptive impact on rehabilitation outcomes and participation. The impact and persistent nature of apathy significantly inhibit engagement in meaningful and daily activities. Participation is thought to be involved in intrinsically motivating occupations that are meaningful, provide individuals a sense of purpose [50], and contribute to their quality of life [6]. Clearly, the key symptom of apathy, that is, diminished motivation [20], negatively affects recovery from stroke and participation in everyday life. More work needs to be done to recognize the presence of apathy in the stroke population, to understand how it may be intertwined with cognitive deficits, and to develop intervention techniques to address both these areas.

## 5. Study Limitations and Recommendations

There were several limitations in this study that warrant the need for future research. First, our outcome measure of participation is a quantitative measure of the retention of prestroke activities. Measuring participation in this manner allows participants to individualize the report of their current participation to their own prior level. However, this measure does not probe their satisfaction in these activities and whether these activities are meaningful for them to resume. Future studies may want to consider supplemental information about satisfaction with participation. Second, our assessment battery largely consisted of questionnaires and pencil-and-paper performance measures. Future studies should consider implementing performance-based functional measures in addition to our paper-and-pencil-based quantitative methods. Third, the mean NIHSS score of our sample was 2.9, indicating mild stroke severity. Our study does not generalize to people who may be living in the community after moderate stroke. Lastly, our cross-sectional design provides information regarding correlations among variables all measured at one time point. Therefore, we are unable to provide a model that predicts which factors measured early in recovery will affect participation later in recovery. Future studies should consider a longitudinal design.

Based on the findings, community-dwelling individuals with milder stroke severity are experiencing decreased participation retention in activities with both low and high cognitive demands. There are barriers and facilitators to poststroke participation in cognitively demanding tasks. Clinicians ought to measure and explain to clients and their caregivers the negative impact of apathy on participation outcomes. For high-CS activities in particular, diminished cognitive abilities will have an impact on participation and may require modifications of the environment or the activities themselves to promote participation. Clinicians should educate clients, family, and friends about the value of social support in poststroke recovery. Loved ones and other persons in the community can provide meaningful influence on poststroke participation by showing up, being present, and available to enjoy activities. Finally, rehabilitation professionals need to recognize that efforts to promote stroke recovery should not stop at recovering independence in activities of daily living and self-care routines but should extend into promoting participation in meaningful activities in the daily life of their clients.

## Figures and Tables

**Table 1:** Demographic characteristics of participations.

Demographic variables	Participants (*N* = 81)
Gender	
Female	42
Male	39
Self-reported race and ethnicity	
Caucasian	43
African American	34
Hispanic/Latino	1
Asian	2
Native American	1
Mean age in years	60
Age range	33-81
Mean time poststroke in months (SD)	57 (78)
Time poststroke range in months	6-360
Education in years (SD)	15 (2.6)
NIH Stroke Scale mean (SD)	2.9 (2.5)
NIH Stroke Scale range	0-10
NIH Stroke Scale Total of Motor Items^∗^	1.0 (1.7)
NIH Stroke Scale Motor Item range	0-8

^∗^NIH Stroke Scale Total Motor Items are the sum of the scores of items 5a and b and 6a and b that grade the extent of motor impairment in the upper extremities and lower extremities on a scale of 0, indicating no impairment, to 4, indicating no movement; total possible score is 16.

**Table 2:** High-cognitive skill activities (*n* = 27).

ACS (number) and activity	ACS domain
(1) Shopping in a store (2) Shopping for groceries (7) Cooking dinner (8) Household maintenance (9) Fixing things around the house (10) Driving (12) Car maintenance (15) Paying bills (16) Managing investment (19) Child care (20) Work (paid) (28) Computer (email, paying bills, shopping) (31) Playing cards (solitaire, poker, bridge) (32) Putting together puzzles (33) Crossword or Sudoku puzzle (37) Playing a musical instrument (38) Reading magazines/books (39) Reading newspaper (40) Reading the bible/religious materials (42) Creative writing/journal (43) Letter writing (57) Playing team sports (58) Woodworking (73) Studying for personal advancement (74) Traveling local/regional (75) Traveling national/international (86) Storytelling with children	IADL IADL IADL IADL IADL IADL IADL IADL IADL IADL IADL LD leisure LD leisure LD leisure LD leisure LD leisure LD leisure LD leisure LD leisure LD leisure LD leisure HD leisure HD leisure Social Social Social Social

IADL: instrumental activities of daily living; LD: low demand (physically); HD: high demand (physically).

**Table 3:** Low-cognitive skill activities (*n* = 14).

ACS (number) and activity	Domain
(17) Resting (18) Beauty/barbershop (21) Spectator sport (44) Birdwatching (46) Going to garden or park (47) Attending concerts (51) Watching movies (52) Watching television (53) Listening to music (54) Listening to radio (55) Sitting and thinking (61) Walking (62) Running (83) Going to a place of worship	IADL IADL LD leisure LD leisure LD leisure LD leisure LD leisure LD leisure LD leisure LD leisure LD leisure HD leisure HD leisure Social

IADL: instrumental activities of daily living; LD: low demand (physically); HD: high demand (physically).

**Table 4:** Percent retained participation by aphasia status.

Outcome variables	PWA mean (SD) *N* = 43	PWOA mean (SD) *N* = 38	All (SD) *N* = 81
% retained high-CS activities	67.9 (23)	63.4 (21)	65.8 (22)
% retained low-CS activities	79.4 (20)	81.3 (17)	80.3 (19)

PWA: persons with aphasia; PWOA: persons without aphasia.

**Table 5:** Pearson correlations of potential predictors with Activity Card Sort outcomes for high-cognitive skill and low-cognitive skill activities.

Predictor variables	% retained low-CS activities	% retained high-CS activities
DKEFS cognition composite	0.273^∗^	0.410^∗∗^
Apathy Evaluation Scale	-0.487^∗∗^	-0.490^∗∗^
MOS-SSS: positive social interaction	0.501^∗∗^	0.498^∗∗^

DKEFS: Delis-Kaplan Executive Function Scales; MOS-SSS: Medical Outcome Study-Social Support Scale; ^∗^*p* < 0.05; ^∗∗^*p* < 0.01.

**Table 6:** Multiple regression results for percent retained participation in low-cognitive skill and high-cognitive skill activities.

Predictor variable	*β*-Weight	*p* value
Low-cognitive skill activities		
DKEFS cognition composite	0.146	0.131
Apathy Evaluation Scale^∗^	-0.279	0.016
MOS-SSS: positive social interaction^∗^	0.316	0.006
*R* ^2^	0.334	
High-cognitive skill activities		
DKEFS cognition composite^∗^	0.292	0.002
Apathy Evaluation Scale^∗^	-0.264	0.016
MOS-SSS: positive social interaction^∗^	0.291	0.008
*R* ^2^	0.394	

DKEFS: Delis-Kaplan Executive Function System; MOS-SSS: Medical Outcome Study-Social Support Scale; ^∗^statistically significant independent predictor.

**Table 7:** Mean scores and standard deviations (in parentheses) for those with and without clinically significant apathy.

Variables	No apathy (SD) *N* = 58	Apathy (SD) *N* = 23
% retained low CS activities^∗∗^	85.6 (14)	69.0 (22)
% retained high CS activities^∗∗^	71.9 (20)	52.9 (21)
MOS-SSS: positive social interaction^∗∗^	85.5 (24)	60.1 (30)

Apathy: Apathy Evaluation Scale score of 37 or greater; ^∗∗^*p* < 0.01.

## Data Availability

Data are available by request to the corresponding author.
